# PCR Array Technology in Biopsy Samples Identifies Up-Regulated mTOR Pathway Genes as Potential Rejection Biomarkers After Kidney Transplantation

**DOI:** 10.3389/fmed.2021.547849

**Published:** 2021-02-17

**Authors:** Isabel Legaz, María Victoria Bernardo, Rafael Alfaro, Helios Martínez-Banaclocha, Jose Antonio Galián, Victor Jimenez-Coll, Francisco Boix, Anna Mrowiec, Diego Salmeron, Carmen Botella, Antonio Parrado, María Rosa Moya-Quiles, Alfredo Minguela, Santiago Llorente, Jesús de la Peña-Moral, Manuel Muro

**Affiliations:** ^1^Department of Legal and Forensic Medicine, Faculty of Medicine, Biomedical Research Institute (IMIB), University of Murcia, Murcia, Spain; ^2^Department of Immunology, University Clinical Hospital Virgen de la Arrixaca-Biomedical Research Institute of Murcia (IMIB), Murcia, Spain; ^3^Departamento de Ciencias Sociosanitarias, Universidad de Murcia, Murcia, Spain; ^4^Centro de Investigación Biomédica en Red (CIBER) Epidemiología y Salud Pública (CIBERESP), Murcia, Spain; ^5^Instituto Murciano de Investigacion Biomédica-Arrixaca, Murcia, Spain; ^6^Department of Nephrology, University Clinical Hospital Virgen de la Arrixaca-Biomedical Research Institute of Murcia (IMIB), Murcia, Spain; ^7^Department of Pathology Services, University Clinical Hospital Virgen de la Arrixaca-Biomedical Research Institute of Murcia (IMIB), Murcia, Spain

**Keywords:** mTOR, Gene expression, medico-legal autopsy, antibody-mediated rejection, PCR array

## Abstract

**Background:** Antibody-mediated rejection (AMR) is the major cause of kidney transplant rejection. The donor-specific human leukocyte antigen (HLA) antibody (DSA) response to a renal allograft is not fully understood yet. mTOR complex has been described in the accommodation or rejection of transplants and integrates responses from a wide variety of signals. The aim of this study was to analyze the expression of the mTOR pathway genes in a large cohort of kidney transplant patients to determine its possible influence on the transplant outcome.

**Methods:** A total of 269 kidney transplant patients monitored for DSA were studied. The patients were divided into two groups, one with recipients that had transplant rejection (+DSA/+AMR) and a second group of recipients without rejection (+DSA/–AMR and –DSA/–AMR, controls). Total RNA was extracted from kidney biopsies and reverse transcribed to cDNA. Human mTOR-PCR array technology was used to determine the expression of 84 mTOR pathway genes. STRING and REVIGO software were used to simulate gene to gene interaction and to assign a molecular function.

**Results:** The studied groups showed a different expression of the mTOR pathway related genes. Recipients that had transplant rejection showed an over-expressed transcript (≥5-fold) of AKT1S1, DDIT4, EIF4E, HRAS, IGF1, INS, IRS1, PIK3CD, PIK3CG, PRKAG3, PRKCB (>12-fold), PRKCG, RPS6KA2, TELO2, ULK1, and VEGFC, compared with patients that did not have rejection. AKT1S1 transcripts were more expressed in +DSA/–AMR biopsies compared with +DSA/+AMR. The main molecular functions of up-regulated gene products were phosphotransferase activity, insulin-like grown factor receptor and ribonucleoside phosphate binding. The group of patients with transplant rejection also showed an under-expressed transcript (≥5-fold) of VEGFA (>15-fold), RPS6, and RHOA compared with the group without rejection. The molecular function of down-regulated gene products such as protein kinase activity and carbohydrate derivative binding proteins was also analyzed.

**Conclusions:** We have found a higher number of over-expressed mTOR pathway genes than under-expressed ones in biopsies from rejected kidney transplants (+DSA/+AMR) with respect to controls. In addition to this, the molecular function of both types of transcripts (over/under expressed) is different. Therefore, further studies are needed to determine if variations in gene expression profiles can act as predictors of graft loss, and a better understanding of the mechanisms of action of the involved proteins would be necessary.

## Introduction

Humoral rejection in renal transplantation is usually caused by the presence of preformed antibodies in the recipient against human leukocyte antigens (HLA) of the donor (1, 2), referred to as donor-specific antibodies (DSAs). Antibody-mediated rejection (AMR) is the major cause of kidney transplant rejection (3–5). The donor-specific human leukocyte antigen (HLA) antibody (DSA) response to a renal allograft is not fully understood (6). Some patients with DSAs develop chronic or acute AMR and eventually reject their allografts, while others do not with biopsies showing normal histopathology (7–10). Therefore, the influence of HLA matching and preformed DSAs in kidney transplantation remains unclear (11–14).

The mammalian target of rapamycin (mTOR) is a conserved large serine/threonine protein kinase and a member of the phosphoinositide-3-kinase (PI3K) related kinase family (15, 16). The mTOR forms two structurally and functionally distinct complexes, called rapamycin-sensitive complex 1 mammalian target 1 (mTORC1) and rapamycin-insensitive complex 2 mammalian target 2 (mTORC2). mTORC1 consists of mTOR, raptor, GβL, and DEPTOR, while mTORC2 consists of mTOR, RICTOR, GβL, PRR5, and SIN1. mTORC1 combines signals from various growth factors, nutrients and energy supply to promote cell growth and regulates multiple biosynthetic cellular processes (protein synthesis, cell cycle progression, cell growth, and proliferation) (17–22). mTORC1 mainly regulates cell growth and metabolism, while mTORC2 controls cell proliferation and survival in particular (17, 20, 22). Nowadays, some mTORC2 regulators have been identified, and although more of them are yet to be discovered, new mechanisms of selectively inhibiting mTORC2 are emerging (23).

Studies involving selective gene regulation of mTOR complexes (mTORC1 and mTORC2) in renal cell populations and/or inhibition of pharmacological mTOR revealed important roles of mTOR in homeostasis of podocytes and tubular transport (24, 25). There have also been important advances in understanding the function of mTOR in kidney injury, polycystic kidney disease and glomerular diseases, including diabetic nephropathy (26–29).

Since mTOR plays a role in the regulation of immune cells metabolism, function, and reactivity (30–32) it has an influence on kidney transplant rejection, glomerulonephritis, and their treatment (19, 33). Both innate and adaptive immune cells (T and B lymphocytes) reside in the kidney and can promote acute and chronic renal disease (34–36), but their dependence on mTOR activity remains unexplored.

An area of interest is the potentially beneficial impact of mTOR inhibition in patients who have performed DSA at the time of transplant (23), because mTOR complex has been described in accommodation or rejection phenomena (37, 38) with non-definite conclusions about these important points (23). The analysis of gene expression of mTOR pathway in kidney biopsies of human transplant could be a biomarker to prevent or anticipate an eventual kidney rejection (39). A study in transplant recipients identified a complex signaling network triggered by HLA II antibody in vascular endothelial cells and indicated that combined ERK and mTORC2 inhibitors may be required to achieve optimal efficacy in controlling HLA II antibody-mediated AMR (40). In addition, several clinical studies of mTORi in heart transplant recipients demonstrated a significant reduction in the progression of cardiac allograft vasculopathy (CAV) and cytomegalovirus (CMV) infection with any mTOR inhibitor regimen, at the expense of higher rates of drug toxicity. Combining an mTOR inhibitor with mycophenolate mofetil (MMF) may also prevent calcineurin inhibitor-induced nephrotoxicity, but this benefit is offset by an increased risk of acute cellular rejection (ACR). Overall mortality rates were not affected by the use of an mTOR inhibitor. These findings in heart transplant may help to design more effective maintenance immunosuppression regimens (41–45).

Therefore, our aim was to explore the relationship between mTOR pathway gene expression and histological and immunological changes in a large cohort of kidney transplant patients to determine if there are differences in the mTOR pathway genes between patients that had kidney rejection (+DSA/+AMR) and patients without rejection (+DSA/–AMR and –DSA) to determine its possible influence on the transplant outcome.

## Methods

### Patient Enrollment and Data Acquisition

A total of 269 adult sequential kidney transplant (KT) patients were recruited at the University Clinic Hospital Virgen de la Arrixaca (Spain) during the period 2015–2019. The clinical, sociodemographic, biochemical data of kidney biopsy of transplant patients were studied. Mean age of total cohort of KT recipients was 46.0 ± 13.2 years (mean ± SD) of which 63% (*n* = 170) were men and 37% (*n* = 99) were women (Table 1). According to data from the Spanish National Transplant Organization (ONT), 59% of kidney recipients are men and 41% are women (46). Transplants were performed using unrelated cadaveric donors.

**Table 1:** Demographic data and main kidney transplantation indications.

**Total of transplantations**	**269**
**Age recipient (mean ± SD)**	
Recipient	46.0 ± 13.2
Donor	51.0 ± 17.5
**Gender, *n* (%)**	
Male	170 (63)
Female	99 (37)
**Transplantation indications, *n* (%)**	
Glomerulonephritis	92 (34.2)
Polycystic kidney disease	55 (20.3)
Type I diabetes mellitus	32 (11.9)
Chronic obstructive pyelonephritis	23 (8.4)
Unknown renal insufficiency	16 (6.1)
Lupic nephritis	10 (3.6)
Reflux nephropathy	6 (2.4)
Others	35 (13.1)
**Transplantation outcome**	
**With rejection**	
+DSA/+AMR	14 (5.2)
**Without rejection**	
+DSA/–AMR	2 (0.7)
–DSA/–AMR	253 (94.1)

*N, number of individuals with a particular disease; AMR, antibody-mediated rejection; DSA, donor specific antibodies. The mean values were analyzed (mean value ± SD) in all cases*.

Estimated glomerular filtration rate (eGFR) and creatinine were analyzed in all transplant patients considering normal values in accordance with the National Kidney Foundation: creatinine 0.7–1.2 mg/dl and eGFR >90 ml/min/1.73m^2^ (47). Our cohort of patients showed the following values before the transplant: creatinine (mg/dl; 2.9 ± 2.1; mean ± SD) and an eGFR <60 ml/min/1.73m^2^ for more than 3 months, which suggests a chronic kidney disease.

Only patients whose kidney graft functioned for at least 1-month post-transplantation and had DSA Luminex determinations for detection of anti-HLA antibodies (T and B cells) screening before transplantation were included in this study. Allograft loss was presumed if patients required dialysis.

Before participating in our study, all patients gave their informed consent to be included as a subject in the investigation. The research was carried out in compliance with the Helsinki Agreement, and the protocol was approved by the HCUVA Ethics Committee (PI15/01370).

### Indications for Kidney Transplant

All of the patients in this study had end-stage kidney disease and were transplanted. As shown in Table 1, the main indication for KTs in our cohort (*n* = 269) was glomerulonephritis (*n* = 92; 34.2%), followed by polycystic kidney disease (*n* = 55; 20.3%), type I diabetes (*n* = 32; 11.9%), chronic obstructive pyelonephritis (*n* = 23; 8.4%), unknown renal insufficiency (*n* = 16; 6.1), lupic nephritis (*n* = 10; 3.6%), reflux nephropathy (*n* = 6; 2.4%). The rest of pathologies were included as others indications (*n* = 35; 13.1%).

### Immunosuppressive Treatment

All enrolled recipients had similar triple immunosuppressive therapy, consisting of oral tacrolimus (Program, Astellas, Ireland), mycophenolate mofetil (MMF; CellCept, Roche, Switzerland), and prednisolone (Dacortin, Merck, Spain) as previously published (48–50).

The starting dose for the Tacrolimus (FK) based protocol was 0.10–0.15 mg/kg/day and the dose was adjusted to maintain an FK level in whole blood between 8 and 12 ng/ml during the first month post-transplant, between 7 and 10 ng/ml during months 2 and 3 post-transplant and between 5 and 8 ng/ml, thereafter. The starting dose for MMF was 2,000 mg/day, and reduced to 1,000–1,500 mg/day during the first month post-transplant, based on white blood cells count.

Methylprednisolone was administered intravenously at doses of 500, 250, and 125 mg/day on the day of transplantation, days 1–2 and days 3–4 after the operation, respectively. Oral prednisolone treatment started on day 5 after the operation with a dose of 20 mg/day, and then tapered to 5–10 mg/day within 2–3 months after transplant. No rapamycin was administered in this group of patients.

### Kidney Rejection Diagnosis

Allograft acute cellular rejection (ACR) was defined as an increase in serum creatinine of at least 20% above baseline serum creatinine and confirmed by biopsy. Protocol biopsies were classically not performed in our clinical hospital. The indication for biopsy was increased creatinine values and/or presence of DSA antibodies in routine evaluation. In the case of patients with DSA+/AMR+ (*n* = 14), the mean age was 45.3 ± 19.2, gender was distributed in 9 males and 5 females, and indications pre-transplant were 6 patients with glomerulonephritis, 3 patients with polycystic kidney disease, 2 patients with diabetes type I, 1 patient with chronic obstructive pyelonephritis, 1 patient with lupic nephritis and one more with reflux nephropathy. The two patients with DSA+/AMR– (*n* = 2) had a mean age of 41.7 ± 21.4, gender was distributed in 1 male and 1 female, and indications pre-transplant were 1 patient with glomerulonephritis and the other one with polycystic kidney disease.

Specimens were evaluated by light microscopy and immunofluorescence staining with a marker of classical complement activation (C4d) and classified according to Banff classification as updated in 2017 (51). The diagnosis of acute antibody-mediated rejection (AMR) requires the presence of distinguishable histopathological findings, a positive C4d staining in peritubular capillaries, and the simultaneous presence of DSA (52). For the renal transplant, a consensus agreement was reached, indicating that a diagnosis of AMR requires the simultaneous presence of DSA, distinguishable histopathological findings and deposition of C4d in peritubular capillaries. Six patients were diagnosed in the first 3-month after kidney transplant, three patients were diagnosed between months 3 and 6, three patients between months 6 and 12, and two patients were diagnosed after the 12th month. Mean serum creatinine at the time of renal biopsy was 3.9 ± 3.7 mg/dL. Proteinuria was 2.97 ± 4.21 g/day. Of the 14 AMR biopsied specimens, 4 presented intimal arteritis. Interstitial inflammation was present in 12 biopsies, tubulitis in 10, glomerulitis in 9, and peritubular capillarity is in 11.

Mild acute cellular rejection (Banff grade I) was treated with pulse steroids (500 mg methylprednisolone boluses) and increased maintenance immunosuppression. All other ACR were treated with anti-thymocyte globulin (ATG).

Acute rejection episodes were further classified as steroid-sensitive rejections (ACR Banff grade I) or steroid-insensitive rejections, ACR Banff grade II and III, and antibody-mediated rejection (AMR). AMR was also treated with pulse steroids and intravenous immunoglobulin (0.25 g/kg) and the last session 1 g/kg (maximum 140 g) divided in two doses associated with plasmapheresis (3 sessions a day, every 5 days). Afterwards, we administered 500 mg anti-CD20 (Rituximab, Roche pharmaceuticals) intravenously. Anti-AMR treatment was also administered in two patients receiving anti-proteasome inhibitor Bortezomic (Velcade®, formerly PS-341). No correlation was observed between acute T-cell mediated rejection (TCMR) and pre- and post-transplant DSA (data not shown).

### Determination of the Causes of Kidney Graft Loss

In the cases of sudden death, the major causes of kidney graft failure have been examined in total patients and obtained from medical death certificates and/or medico-legal autopsy to determine the cause and circumstances of death.

### DSAs Luminex anti-HLA Antibody Screening

DSAs Luminex anti-HLA antibody test was performed in serum collected every 3 months from all the patients in this study while on the waiting list. The time points for dynamic testing post-transplantation were established and serum samples were obtained at week 2, at months 1, 3, 6, and 12 and then annually for 3 years, and when clinically indicated. All serum samples were analyzed and tested for anti-DSA using microbeads solid phase luminex-based SAB (OneLambda, CA).

Antibody screening by multiplex Luminex was performed in all samples (LABScreen® Mix and SAB kits, OL, CA). Color-coded microspheres, coated with the major HLA class I and II antigens, were incubated with the serum for 30 min at room temperature in the dark. After three washes the samples were incubated with 100 μL of 1:100 phycoerythrin-conjugated goat anti-human IgG (One Lambda). Finally, after two washes, the fluorescent signal intensity for each microsphere was measured using LABScan® 100 Flow analyzer (Luminex, Austin, TX). The cut-off for positive samples was the Normalized Background (NBG) ratio as recommended by the manufacturer and it was obtained with HLA Fusion® software 4.0 (One Lambda).

The reporter fluorescence intensity of each bead was expressed as median fluorescence intensity (MFI) levels which is directly proportional to the amount of antibody bound to the microspheres. MFI levels higher than 1,000–1,500 are usually considered positive, as commonly accepted (53, 54). *De novo* DSA (dnDSA) was considered positive if new IgG antibodies not present at the time of transplantation were detected and the normalized intensity *via* single antigen bead was 1,500 MFI. We also tested to what extent prozone might have masked the presence of allo-antibodies prior to transplant and post-transplant for DTT or EDTA treatment and also tested neat and 1:8 and 1:16 titer in parallel and all results were similar.

### Isolation of RNA and cDNA Synthesis

Total RNA was extracted and isolated from kidney biopsies after tissue homogenization (Trizol; Invitrogen, Carlsbad, CA) and then purified by RNeasy®MinElute™ Purification Kit (Qiagen, Dusseldorf, Germany). Genomic DNA (gDNA) was removed by treatment with DNase I (Sigma Aldrich), according to the manufacturer's instructions and reverse transcribed to cDNA using a RT^2^ First Strand Kit (SABiosciences, Qiagen) and stored at −20°C.

The absence of contaminating gDNA was demonstrated by the absence of a product in wells using “no RT” control samples, which included all components of the cDNA synthesis reaction, except for reverse transcripts. The quality and concentration of RNA was assessed by spectrophotometry (NanoDrop 2000/2000c Thermofisher Scientific). RNA integrity was assessed by electrophoresis of the denaturing agarose gel. For each RNA sample thick bright bands of 28s and 18s ribosomal RNA were observed, indicating that the RNA integrity was appropriate. The concentration of extracted RNA ranged from 369 to 612 ng/μL, with OD260/280 ratios ranging from 1.8 to 2.1, in accordance with experimental requirements.

### Gene Expression of mTOR Pathway

The expression of a total of 84 key genes involved in the human mTOR pathway (RT^2^ Profiler™ PCR Array Human mTOR Signaling, Qiagen, Germany, Cod. PAHS098Z) was analyzed in kidney biopsies according to the manufacturer's protocol. Gene expression assays analyzed are listed in Table 2, which shows the access code to the GenBank (47) and UniGene (48) data bases and were used according to the manufacturer's instructions in triplicates.

**Table 2:** Summary of 84 human mTOR pathway related genes analyzed by qPCR array in the kidney biopsies.

**N^**°**^**	**Gene symbol**	**Gene name**	Databases*	
			**GenBank**	**UniGene**
1	AKT1	V-akt murine thymoma viral oncogene homolog 1	NM_005163	Hs.525622
2	AKT1S1	AKT1 substrate 1 (proline-rich)	NM_032375	Hs.515542
3	AKT2	V-akt murine thymoma viral oncogene homolog 2	NM_001626	Hs.631535
4	AKT3	VAKT homolog 3 (protein kinase B, gamma)	NM_005465	Hs.498292
5	CAB39	Calcium binding protein 39	NM_016289	Hs.632536
6	CAB39L	Calcium binding protein 39-like	NM_030925	Hs.87159
7	CDC42	Cell division cycle 42 (GTP binding protein, 25 kDa)	NM_001791	Hs.690198
8	CHUK	Conserved helix-loop-helix ubiquitous kinase	NM_001278	Hs.198998
9	DDIT4	DNA-damage-inducible transcript 4	NM_019058	Hs.523012
10	DDIT4L	DNA-damage-inducible transcript 4-like	NM_145244	Hs.480378
11	DEPTOR	DEP domain containing MTOR-interacting protein	NM_022783	Hs.112981
12	EIF4B	Eukaryotic translation initiation factor 4B	NM_001417	Hs.648394
13	EIF4E	Eukaryotic translation initiation factor 4E	NM_001968	Hs.249718
14	EIF4EBP1	Eukaryotic translation initiation factor 4E binding protein 1	NM_004095	Hs.411641
15	EIF4EBP2	Eukaryotic translation initiation factor 4E binding protein 2	NM_004096	Hs.730236
16	FKBP1A	FK506 binding protein 1A, 12 kDa	NM_000801	Hs.471933
17	FKBP8	FK506 binding protein 8, 38 kDa	NM_012181	Hs.173464
18	GSK3B	Glycogen synthase kinase 3 beta	NM_002093	Hs.445733
19	HIF1A	Hypoxia inducible factor 1, alpha subunit (basic helix-loop-helix transcription factor)	NM_001530	Hs.597216
20	HRAS	V-Ha-ras Harvey rat sarcoma viral oncogene homolog	NM_005343	Hs.37003
21	HSPA4	Heat shock 70 kDa protein 4	NM_002154	Hs.90093
22	IGF1	Insulin-like growth factor 1 (somatomedin C)	NM_000618	Hs.160562
23	IGFBP3	IGF binding protein 3	NM_000598	Hs.450230
24	IKBKB	Inhibitor of kappa light polypeptide gene enhancer in B-cells, kinase beta	NM_001556	Hs.597664
25	ILK	Integrin-linked kinase	NM_004517	Hs.5158
26	INS	Insulin	NM_000207	Hs.654579
27	INSR	Insulin receptor	NM_000208	Hs.465744
28	IRS1	Insulin receptor substrate 1	NM_005544	Hs.471508
29	MAPK1	Mitogen-activated protein kinase 1	NM_002745	Hs.431850
30	MAPK3	Mitogen-activated protein kinase 3	NM_002746	Hs.861
31	MAPKAP1	Mitogen-activated protein kinase associated protein 1	NM_024117	Hs.495138
32	MLST8	MTOR associated protein, LST8 homolog (*S. cerevisiae*)	NM_022372	Hs.29203
33	MTOR	Mechanistic target of rapamycin (serine/threonine kinase)	NM_004958	Hs.338207
34	MYO1C	Myosin IC	NM_033375	Hs.286226
35	PDPK1	3-phosphoinositide dependent protein kinase-1	NM_002613	Hs.459691
36	PIK3C3	Phosphoinositide-3-kinase, class 3	NM_002647	Hs.464971
37	PIK3CA	Phosphoinositide-3-kinase, catalytic, alpha polypeptide	NM_006218	Hs.553498
38	PIK3CB	Phosphoinositide-3-kinase, catalytic, beta polypeptide	NM_006219	Hs.239818
39	PIK3CD	Phosphoinositide-3-kinase, catalytic, delta polypeptide	NM_005026	Hs.518451
40	PIK3CG	Phosphoinositide-3-kinase, catalytic, gamma polypeptide	NM_002649	Hs.32942
41	PLD1	Phospholipase D1, phosphatidylcholine-specific	NM_002662	Hs.382865
42	PLD2	Phospholipase D2	NM_002663	Hs.104519
43	PPP2CA	Protein phosphatase 2, catalytic subunit, alpha isozyme	NM_002715	Hs.483408
44	PPP2R2B	Protein phosphatase 2, regulatory subunit B, beta	NM_181678	Hs.655213
45	PPP2R4	Protein phosphatase 2A activator, regulatory subunit 4	NM_021131	Hs.400740
46	PRKAA1	Protein kinase, AMP-activated, alpha 1 catalytic subunit	NM_006251	Hs.43322
47	PRKAA2	Protein kinase, AMP-activated, alpha 2 catalytic subunit	NM_006252	Hs.437039
48	PRKAB1	Protein kinase, AMP-activated, beta 1 non-catalytic subunit	NM_006253	Hs.715515
49	PRKAB2	Protein kinase, AMP-activated, beta 2 non-catalytic subunit	NM_005399	Hs.50732
50	PRKAG1	PRK, AMP-activated, gamma 1 non-catalytic subunit	NM_002733	Hs.530862
51	PRKAG2	PRK, AMP-activated, gamma 2 non-catalytic subunit	NM_016203	Hs.647072
52	PRKAG3	PRK, AMP-activated, gamma 3 non-catalytic subunit	NM_017431	Hs.591634
53	PRKCA	Protein kinase C, alpha	NM_002737	Hs.531704
54	PRKCB	Protein kinase C, beta	NM_002738	Hs.460355
55	PRKCE	Protein kinase C, épsilon	NM_005400	Hs.580351
56	PRKCG	Protein kinase C, gamma	NM_002739	Hs.631564
57	PTEN	Phosphatase and tensinhomolog	NM_000314	Hs.500466
58	RHEB	Ras homolog enriched in brain	NM_005614	Hs.283521
59	RHOA	Ras homolog gene family, member A	NM_001664	Hs.247077
60	RICTOR	RPTOR independent companion of MTOR, complex 2	NM_152756	Hs.407926
61	RPS6	Ribosomal protein S6	NM_001010	Hs.408073
62	RPS6KA1	Ribosomal protein S6 kinase, 90 kDa, polypeptide 1	NM_002953	Hs.149957
63	RPS6KA2	Ribosomal protein S6 kinase, 90 kDa, polypeptide 2	NM_021135	Hs.655277
64	RPS6KA5	Ribosomal protein S6 kinase, 90 kDa, polypeptide 5	NM_004755	Hs.510225
65	RPS6KB1	Ribosomal protein S6 kinase, 70 kDa, polypeptide 1	NM_003161	Hs.463642
66	RPS6KB2	Ribosomal protein S6 kinase, 70 kDa, polypeptide 2	NM_003952	Hs.534345
67	RPTOR	Regulatory associated protein of MTOR, complex 1	NM_020761	Hs.133044
68	RRAGA	Ras-related GTP binding A	NM_006570	Hs.432330
69	RRAGB	Ras-related GTP binding B	NM_006064	Hs.50282
70	RRAGC	Ras-related GTP binding C	NM_022157	Hs.532461
71	RRAGD	Ras-related GTP binding D	NM_021244	Hs.31712
72	SGK1	Serum/glucocorticoid regulated kinase 1	NM_005627	Hs.510078
73	STK11	Serine/threonine kinase 11	NM_000455	Hs.515005
74	STRADB	STE20-related kinase adaptor beta	NM_018571	Hs.652338
75	TELO2	TEL2, telomere maintenance 2, homolog (*S. cerevisiae*)	NM_016111	Hs.271044
76	TP53	Tumor protein p53	NM_000546	Hs.654481
77	TSC1	Tuberous sclerosis 1	NM_000368	Hs.370854
78	TSC2	Tuberous sclerosis 2	NM_000548	Hs.90303
79	ULK1	Unc-51-like kinase 1 (*C. elegans*)	NM_003565	Hs.47061
80	ULK2	Unc-51-like kinase 2 (*C. elegans*)	NM_014683	Hs.168762
81	VEGFA	Vascular endothelial growth factor A	NM_003376	Hs.73793
82	VEGFB	Vascular endothelial growth factor B	NM_003377	Hs.78781
83	VEGFC	Vascular endothelial growth factor C	NM_005429	Hs.435215
84	YWHAQ	Tyrosine 3-monooxygenase/tryptophan 5-monooxygenase activation protein, theta polypeptide	NM_006826	Hs.74405

* *Access code to the GenBank (46) and UniGene (47) databases are showed*.

The cDNA template was applied to the quantitative reaction mixture in real time after sufficient dilution, and equal quantities of reaction liquid were applied to each well of the PCR array, containing gene-specific primers. PCR amplification was performed using real-time PCR detection (ABI StepOneplus™, Applied Biosystems, USA) under the following conditions; denaturing at 95°C for 10 min, 40 amplification cycles of denaturation at 95°C for 10 s, and annealing and extension at 60°C for 1 min, followed by acquisition of fluorescence signal. The mean Ct values were calculated from technical triplicates.

Data was analyzed using the comparative ΔΔCt method and expressed as fold-changes (40) in the target gene normalized to the mean of endogenous control genes (ACTB, B2M, GAPDH, HPRT, and RPLP0) in kidney biopsies with and without rejection. In addition to this, human genomic DNA contamination (HGDC), three RTC (Reverse Transcription Control) and three positive PCR Control (PPC) were also used according to the manufacturer's protocol. A gene was considered over or under expressed when there was fivefold (≥5-fold) difference between +DSA/+AMR patients and patients with kidney transplant showing +DSA/–AMR and –DSA samples (40).

Gene expression was analyzed in recipients who presented DSAs (+DSA) and showed anomalous biopsy histopathology and developed AMR (+AMR; *n* = 14) or presented *de novo* DSA (+DSA) without AMR development (+DSA/–AMR; *n* = 2). Biopsies from other recipients that did not have rejection were also analyzed as controls (–AMR; *n* = 253) (Table 1).

### Genetic Relations and Molecular Functions

**Figure 1:**
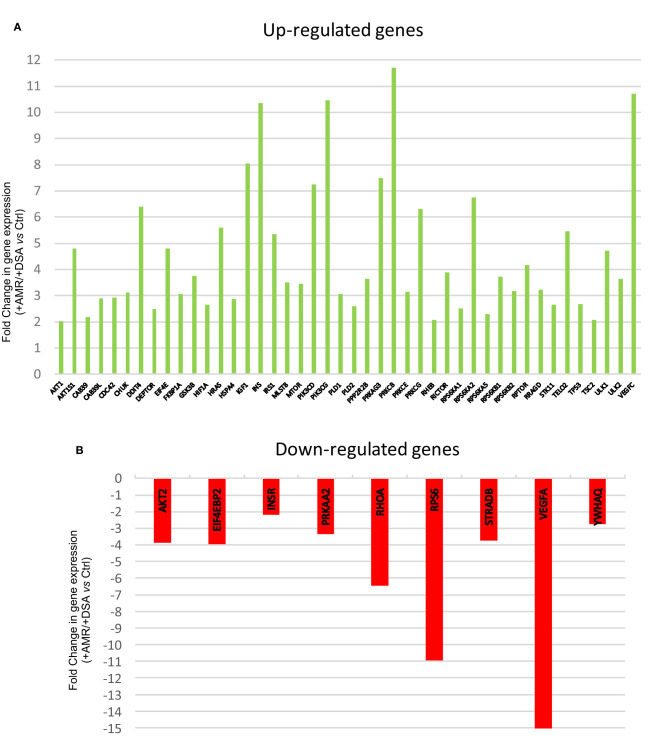
Differentially regulated mTOR pathway genes. **(A)** Up-regulated mTOR pathway genes **(B)** Down-regulated mTOR pathway genes.

**Figure 2:**
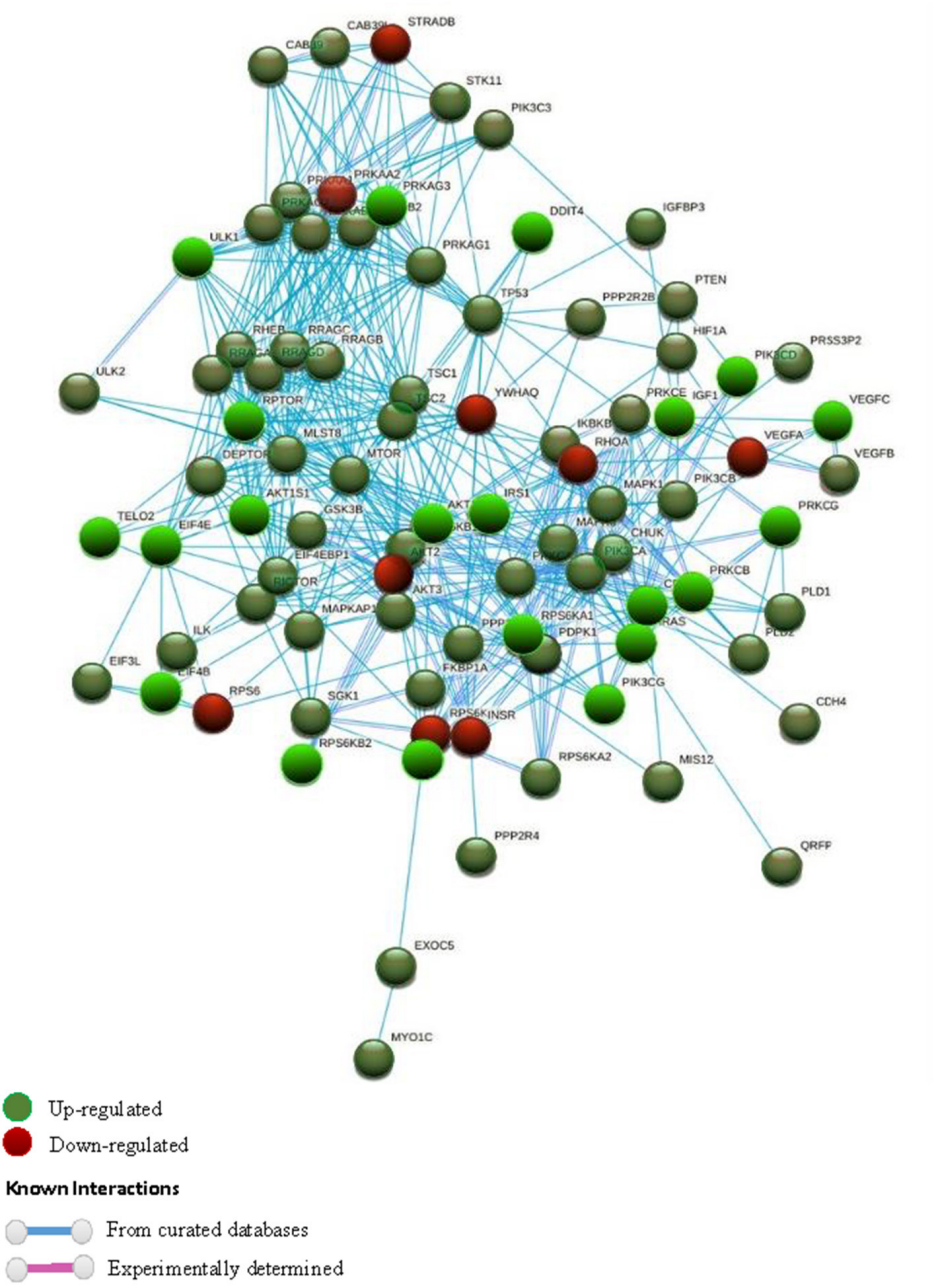
STRING interaction pathway of differentially expressed mTOR pathway genes. Green nodes indicate up-regulated genes and red nodes down-regulated genes comparing +AMR/+DSA and +DSA vs. –AMR/–DSA KT patients. The intensity of the color indicates the degree of regulation of the gene. The blue line shows that the interaction is checked against curated databases and the purple line shows that the interaction is experimentally determined.

STRING software (version 10.5) (55) was used to study the different gene to gene relationships in the two groups of compared biopsies (rejection vs. without rejection; Figures 1, 2). The thickness of the edges of the network represents the strength of the interaction. The required interaction score was set at “the highest confidence” (0.900). Known interactions were obtained from curated or experimentally determined databases and shown with different color lines (blue and purple, respectively). Green circles represented up-regulated genes and red circles represented down-regulated genes (red circles). The most intense colored represents up- or down- expressed transcript (≥5-fold).

**Figure 3:**
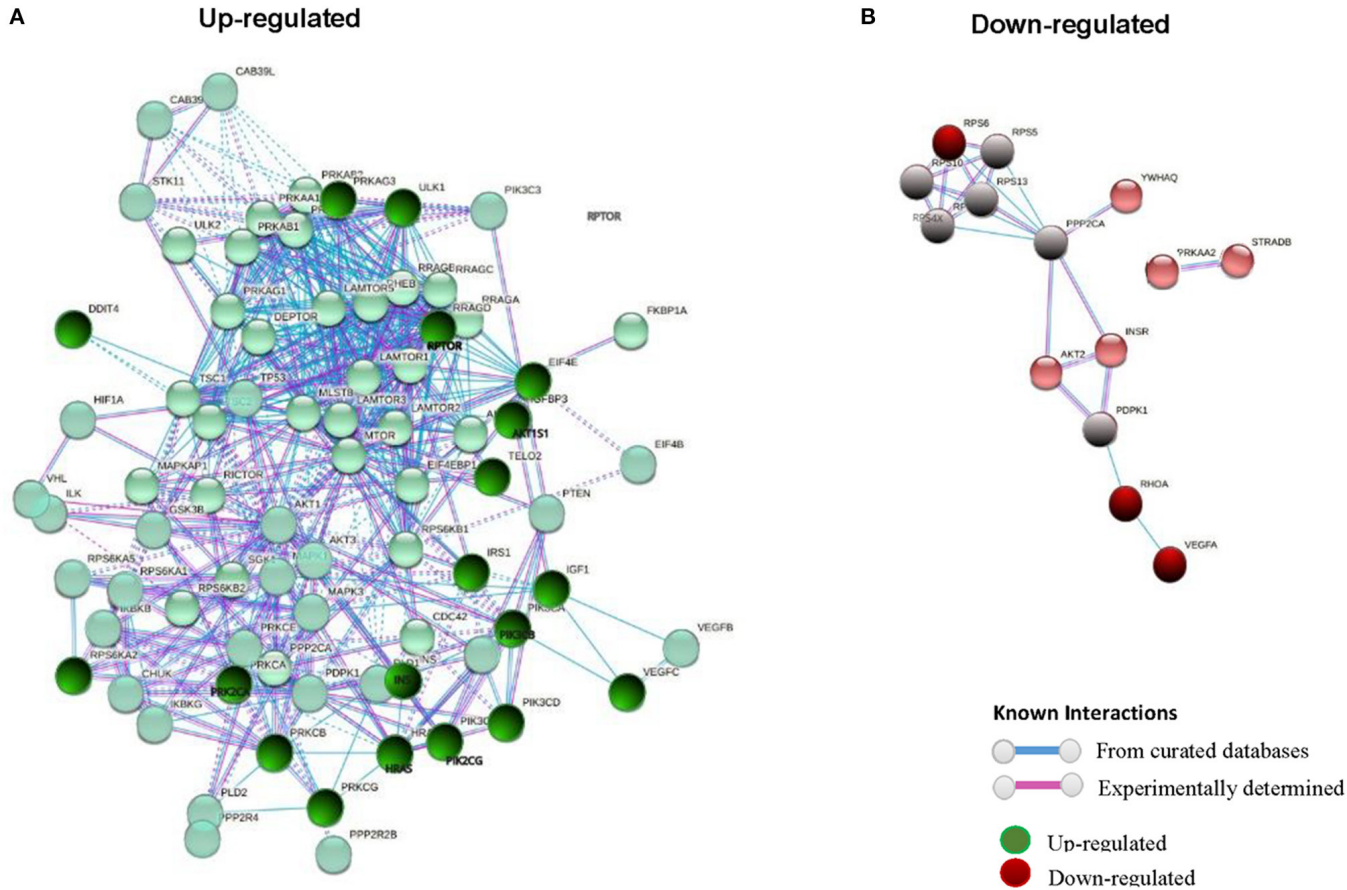
STRING interaction pathway of differentially expressed mTOR pathway genes comparing +AMR/+DSA and +DSA vs. –AMR/–DSA KT patients. **(A)** Green nodes indicate up-regulated genes. **(B)** Red nodes indicate down-regulated genes. The intensity of the color indicates the degree of regulation of the gene. The blue line shows that the interaction is checked against from curated databases and the purple line shows that the interaction is experimentally determined.

**Figure 4:**
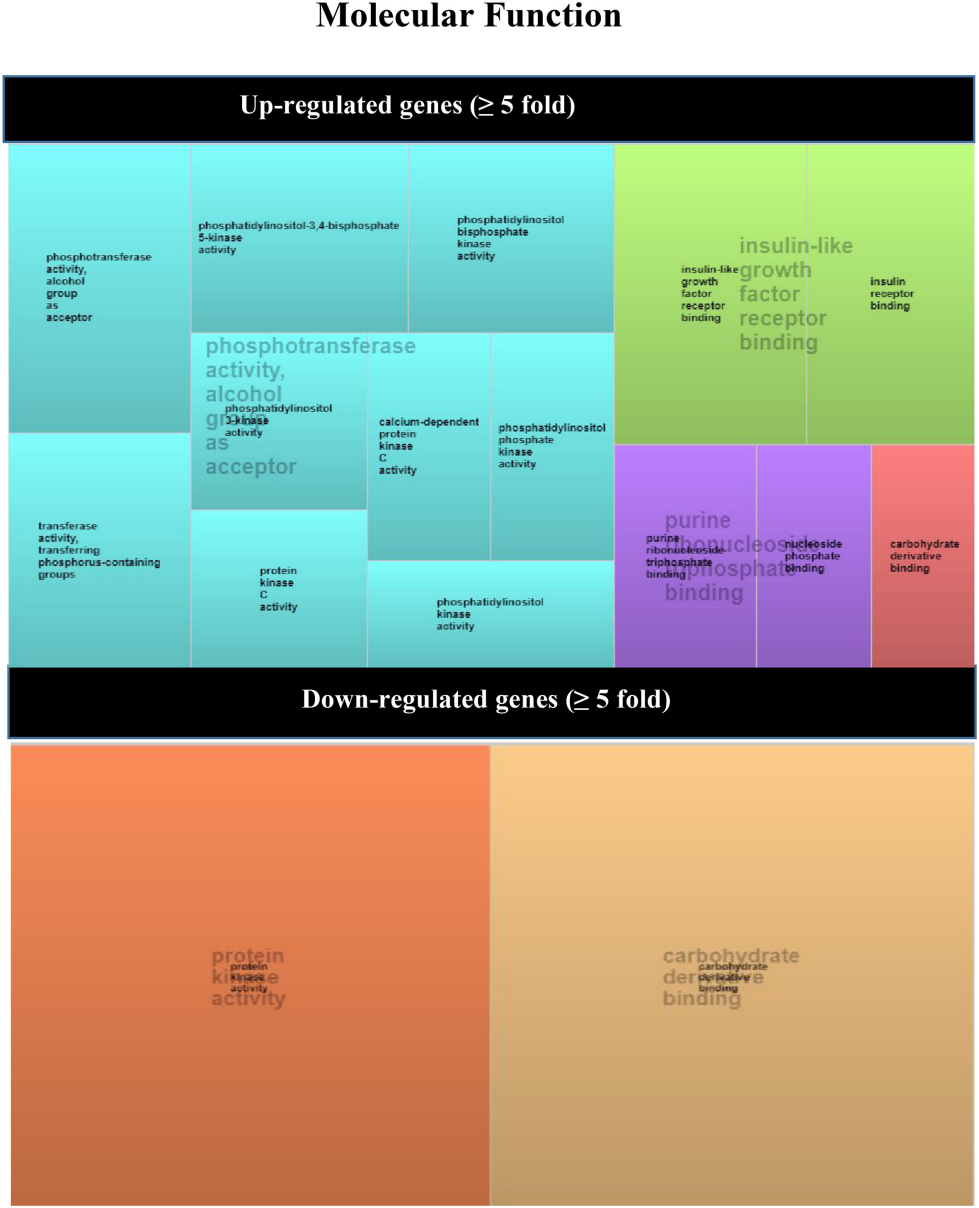
REVIGO TreeMap view of GO terms enriched between up- (upper TreeMaps) or down-regulated (lower TreeMaps) genes in biopsies from +DSA/+AMR patients vs. control recipients (–DSA/–AMR, +DSA/–AMR, and –DSA). Each rectangle is a single cluster representative. The representatives are joined into “superclusters” of loosely related terms that are visualized with distinct colors (indicated by centralized black text). The size of the rectangles reflects the enrichment of the GO term.

REVIGO software (56) was used to assign a molecular function of up- or down-expressed transcript (≥5-fold). The parameters were as follows: allowed similarity = Small (0.5); Homo sapiens database; and Simrel as semantic similarity measure (Figure 3). KEGG mTOR pathway genes were used to locate genes expressed differently (Figure 4).

### Statistical Analysis

Demographic data and results were collected in a database (Microsoft Access 2.0; Microsoft Corporation, Seattle, WA) and the analysis was performed using SPSS 23.0 (SPSS software Inc., Chicago, IL). All results were expressed as the mean ± SD or as a percentage. Demographic, clinical, immunological features, and post-transplant anti-HLA antibodies status were compared using Pearson χ^2^-test or Fisher's exact test for categorical data and Student *t*-test or Mann Whitney *U*-test for continuous data, as appropriate. A two-sided *P*-value <0.05 was considered as statistically significant. The statistical power to detect differential expression of each of the analyzed genes was performed, obtaining a total statistical power of 0.8 (80%) (57).

## Results

### Up-Regulated mTOR Genes in Rejection Kidney Biopsies

The genetic expression of the mTOR pathway in renal biopsies of +DSA/+AMR, +DSA/–AMR, and –DSA/–AMR kidney biopsies were analyzed (Table 3, Figures 1, 2, 3A).

**Table 3:** Genetic expression of the mTOR pathway in renal biopsies of recipients without rejection used as controls and biopsies from rejected transplants.

**Genes**	**Average Ct**		**Average Delta Ct**		**2**^****∧****^**[-Avg.(Delta(Ct))]**		Fold-change*	*p*-value*	Fold regulation*
	**Control**	**Group 1**	**Control**	**Group 1**	**Control**	**Group 1**			
AKT1	35.67	37.86	2.149618	1.140615	0.225372	0.453566	2.0125	0.18523	2.0125
AKT1S1	38.95	39.89	5.430249	3.169554	0.023192	0.11114	4.7922	0.191113	4.7922
AKT2	32.15	37.3	−1.372164	0.579572	2.588585	0.669162	0.2585	0.107187	−3.8684
AKT3	34.65	38.16	1.125971	1.439249	0.458193	0.368759	0.8048	0.574766	−1.2425
CAB39	36.39	38.45	2.864434	1.731576	0.137315	0.301123	2.1929	0.244315	2.1929
CAB39L	36.42	38.09	2.901263	1.367099	0.133854	0.38767	2.8962	0.495093	2.8962
CDC42	35.17	36.82	1.648762	0.101859	0.318914	0.931832	2.9219	0.372582	2.9219
CHUK	36.43	37.99	2.907095	1.270974	0.133314	0.41438	3.1083	0.446243	3.1083
DDIT4	38.94	39.46	5.421603	2.744033	0.023331	0.149267	6.3978	0.389981	6.3978
DDIT4L	36.67	39.11	3.146842	2.392757	0.112903	0.190418	1.6866	0.899678	1.6866
DEPTOR	35.42	37.29	1.896134	0.575323	0.268662	0.671136	2.4981	0.973631	2.4981
EIF4B	34.15	36.63	0.624149	−0.09031	0.648802	1.064599	1.6409	0.692843	1.6409
EIF4E	37.42	38.36	3.902091	1.642677	0.066889	0.320262	4.788	0.31073	4.788
EIF4EBP1	36.91	39.48	3.39283	2.765633	0.095204	0.147049	1.5446	0.721489	1.5446
EIF4EBP2	32.89	38.08	−0.62605	1.358519	1.543334	0.389982	0.2527	**0.005744**	−3.9574
FKBP1A	36.94	38.52	3.415945	1.796989	0.093691	0.287775	3.0715	0.358526	3.0715
FKBP8	35.42	38.67	1.903697	1.946834	0.267258	0.259385	0.9705	0.644696	−1.0304
GSK3B	36.43	37.72	2.907091	1.001307	0.133315	0.499547	3.7471	0.394209	3.7471
HIF1A	35.93	37.72	2.405051	0.999699	0.188802	0.500104	2.6488	0.520589	2.6488
HRAS	37.95	38.66	4.428664	1.943606	0.046434	0.259966	5.5986	0.361074	5.5986
HSPA4	35.69	37.36	2.169912	0.64659	0.222224	0.638789	2.8745	0.360342	2.8745
IGF1	38.4	38.59	4.876377	1.867525	0.034046	0.274043	8.0492	0.277681	8.0492
IGFBP3	34.14	37.43	0.621437	0.711764	0.650023	0.610573	0.9393	0.609412	−1.0646
IKBKB	35.4	38.45	1.875704	1.732641	0.272494	0.300901	1.1042	0.696416	1.1042
ILK	35.17	38.3	1.645954	1.582463	0.319535	0.333911	1.045	0.790086	1.045
INS	40	39.82	6.478997	3.104962	0.011211	0.116229	10.3678	0.15873	10.3678
INSR	34.14	38.46	0.622975	1.742113	0.649331	0.298932	0.4604	0.645888	−2.1722
IRS1	38.7	39.48	5.180884	2.758797	0.027568	0.147747	5.3595	0.369636	5.3595
MAPK1	34.89	38.23	1.366651	1.512592	0.38779	0.350481	0.9038	0.623096	−1.1065
MAPK3	35.67	37.94	2.146867	1.223289	0.225802	0.428305	1.8968	0.576047	1.8968
MAPKAP1	34.4	37.64	0.88135	0.919579	0.542859	0.528663	0.9738	0.594507	−1.0269
MLST8	36.92	38.3	3.396966	1.583056	0.094932	0.333774	3.5159	0.426619	3.5159
MTOR	36.67	38.07	3.145964	1.353567	0.112972	0.391323	3.4639	0.52955	3.4639
MYO1C	33.15	36.99	−0.368758	0.271379	1.291241	0.828527	0.6417	0.996003	−1.5585
PDPK1	34.89	38.54	1.370937	1.821182	0.38664	0.282989	0.7319	0.597437	−1.3663
PIK3C3	35.64	38.59	2.122182	1.87655	0.229699	0.272334	1.1856	0.504634	1.1856
PIK3CA	36.67	39.63	3.149926	2.90728	0.112662	0.133297	1.1832	0.927941	1.1832
PIK3CB	35.18	38.68	1.658021	1.965429	0.316874	0.256063	0.8081	0.982749	−1.2375
PIK3CD	38.9	39.24	5.378186	2.522382	0.024044	0.174055	7.2391	0.486831	7.2391
PIK3CG	39.22	39.03	5.702613	2.31658	0.019202	0.200743	10.4544	0.132621	10.4544
PLD1	36.95	38.52	3.42452	1.804047	0.093136	0.28637	3.0748	0.138445	3.0748
PLD2	36.92	38.73	3.400398	2.015433	0.094706	0.24734	2.6117	0.973547	2.6117
PPP2CA	34.89	37.79	1.36747	1.073546	0.38757	0.47515	1.226	0.540427	1.226
PPP2R2B	37.42	38.75	3.899045	2.029452	0.06703	0.244948	3.6543	0.489087	3.6543
PPP2R4	35.4	38.59	1.879166	1.871819	0.271841	0.273229	1.0051	0.808126	1.0051
PRKAA1	34.64	37.93	1.116091	1.216605	0.461342	0.430294	0.9327	0.566996	−1.0722
PRKAA2	34.39	39.33	0.869814	2.607112	0.547217	0.164127	0.2999	**0.017273**	−3.3341
PRKAB1	34.41	37.79	0.887777	1.075984	0.540446	0.474347	0.8777	0.745967	−1.1393
PRKAB2	36.18	39.11	2.656225	2.394832	0.158634	0.190144	1.1986	0.524063	1.1986
PRKAG1	35.89	38.75	2.372589	2.027652	0.193099	0.245254	1.2701	0.907542	1.2701
PRKAG2	35.41	38.37	1.893278	1.654811	0.269195	0.317579	1.1797	0.530421	1.1797
PRKAG3	39.25	39.54	5.731223	2.823425	0.018825	0.141275	7.5047	0.384654	7.5047
PRKCA	33.89	37.49	0.366599	0.775646	0.775609	0.584127	0.7531	0.867045	−1.3278
PRKCB	36.9	36.55	3.38099	−0.166596	0.095989	1.122407	11.6931	0.285235	11.6931
PRKCE	36.68	38.23	3.15859	1.509198	0.111988	0.351306	3.137	0.349558	3.137
PRKCG	39.23	39.77	5.709559	3.048438	0.01911	0.120873	6.3252	0.230953	6.3252
PTEN	34.15	38.32	0.625454	1.603646	0.648216	0.329044	0.5076	0.850737	−1.97
RHEB	36.67	38.81	3.146609	2.093422	0.112921	0.234324	2.0751	0.461328	2.0751
RHOA	30.9	36.78	−2.624579	0.062383	6.167045	0.957681	0.1553	0.926912	−6.4396
RICTOR	37.2	38.43	3.679059	1.715291	0.078072	0.304541	3.9008	0.125264	3.9008
RPS6	29.15	35.8	−4.373158	−0.922783	20.722964	1.895768	0.0915	**0.012523**	−10.9312
RPS6KA1	36.41	38.28	2.887278	1.559379	0.135158	0.339297	2.5104	0.426115	2.5104
RPS6KA2	37.91	38.35	4.384495	1.629768	0.047878	0.32314	6.7493	0.091908	6.7493
RPS6KA5	37.17	39.18	3.648458	2.457175	0.079745	0.182103	2.2836	0.477513	2.2836
RPS6KB1	35.92	37.22	2.400954	0.498529	0.189339	0.707828	3.7384	0.597609	3.7384
RPS6KB2	37.43	38.96	3.908267	2.238024	0.066603	0.211977	3.1827	0.537535	3.1827
RPTOR	38.2	39.33	4.674328	2.616594	0.039164	0.163052	4.1633	0.404183	4.1633
RRAGA	35.91	38.29	2.39136	1.57228	0.190603	0.336277	1.7643	0.321376	1.7643
RRAGB	35.65	38.73	2.128129	2.012434	0.228754	0.247855	1.0835	0.816654	1.0835
RRAGC	35.42	37.64	1.898919	0.924812	0.268144	0.526749	1.9644	0.357853	1.9644
RRAGD	37.43	38.93	3.908038	2.215852	0.066614	0.215259	3.2315	0.354061	3.2315
SGK1	34.9	37.8	1.374609	1.076748	0.385657	0.474096	1.2293	0.565313	1.2293
STK11	37.95	39.74	4.432971	3.018055	0.046296	0.123445	2.6664	0.219861	2.6664
STRADB	33.15	38.24	−0.374137	1.520769	1.296064	0.3485	0.2689	0.31349	−3.719
TELO2	38.92	39.67	5.399177	2.952239	0.023697	0.129207	5.4526	0.294516	5.4526
TP53	37.19	38.97	3.670369	2.250594	0.078543	0.210138	2.6754	0.067846	2.6754
TSC1	34.89	37.71	1.366215	0.995182	0.387908	0.501672	1.2933	0.616752	1.2933
TSC2	37.17	39.33	3.653478	2.608525	0.079468	0.163967	2.0633	0.487885	2.0633
ULK1	37.7	38.66	4.1821	1.94091	0.055089	0.260452	4.7279	0.438805	4.7279
ULK2	37.7	39.03	4.1749	2.314215	0.055364	0.201072	3.6318	0.470281	3.6318
VEGFA	30.15	37.3	−3.372039	0.57812	10.353447	0.669836	0.0647	**0.002013**	−15.4567
VEGFB	35.16	38.81	1.63432	2.094631	0.322122	0.234128	0.7268	0.763379	−1.3758
VEGFC	38.97	38.75	5.45371	2.032722	0.022818	0.244394	10.7108	0.489772	10.7108
YWHAQ	31.89	36.55	−1.632133	−0.165442	3.099711	1.12151	0.3618	0.144955	−2.7639
ACTB	33.14	36.89	−0.37831	0.172707	1.299818	0.887177	0.6825	0.458808	−1.4651
B2M	31.15	36.29	−2.367976	−0.425856	5.162163	1.343369	0.2602	0.573033	−3.8427
GAPDH	33.9	36.55	0.37831	−0.172707	0.769338	1.127171	1.4651	0.166227	1.4651
HPRT1	36.93	38.81	3.406508	2.088295	0.094306	0.235158	2.4936	0.281297	2.4936
RPLP0	32.9	36.05	−0.620491	−0.666178	1.537398	1.586863	1.0322	0.605154	1.0322
HGDC	39.47	38.44	5.952466	1.726717	0.016148	0.302139	18.7101	0.249236	18.7101
RTC	25.88	27.96	−7.63915	−8.760246	199.3486	433.607572	2.1751	0.860772	2.1751
RTC	25.88	27.82	−7.637846	−8.902247	199.168543	478.457426	2.4023	0.689459	2.4023
RTC	25.88	27.88	−7.643027	−8.834321	199.885125	456.45253	2.2836	0.805091	2.2836
PPC	23.14	22.89	−10.382546	−13.830544	1334.928193	14568.28863	10.9132	0.303848	10.9132
PPC	23.15	23.1	−10.373234	−13.614101	1326.339391	12538.70969	9.4536	0.315517	9.4536
PPC	23.39	23.25	−10.128558	−13.469552	1119.437046	11343.29303	10.133	0.335258	10.133

*Avg, Average; HGDC, Human genomic DNA contamination; RTC, Reverse Transcription Control; PPC, positive PCR control. Endogenous control genes (ACTB, B2M, GAPDH, HPRT and RPLP0). Control group without rejection (–DSA/–AMR and +DSA/–AMR). Group 1, Rejection renal biopsies (Group 1; +DSA/+AMR)*.

* *Rejection renal biopsies comparing to control group. Bold values are considered as statistically significant*.

No statistically significant differences were observed in mTOR pathway gene expression in recipients with no rejection with or without DSA (–DSA/–AMR and +DSA/–AMR; data not shown). Therefore, these biopsies were used as controls and were compared with biopsies from rejected transplants (+DSA/+AMR). A confounder analysis on gene expression levels of mTOR genes and months post-transplantation also resulted in a *p*-value of 0.46, demonstrating post-transplantation time was not a factor driving gene expression values.

A higher number of over-expressed genes of mTOR pathway were found in biopsies from rejected kidney transplants from +DSA/+AMR patients. In the analyzed biopsies, differential expression of the mTOR pathway related genes was detected. As shown in Figure 1, the AKT1S1, DDIT4, EIF4E, HRAS, IGF1, INS, IRS1, PIK3CD, PIK3CG, PRKAG3, PRKCB, PRKCG, RPS6KA2, RPTOR, TELO2, ULK1, VEGFC were over-expressed (≥5-fold) in +DSA/+AMR patients compared with patients with kidney transplant showing +DSA/–AMR and –DSA samples.

However, the following genes did not show significant differences with respect to the controls INS, PIK3CG, PIK3CD, PRKCG, PRKCB, VEGFC despite being over-expressed. Up-regulated mTOR pathway genes are showed in Figure 4.

### Interaction Pathway Up-Regulated mTOR Genes in Rejection Kidney Biopsies

In order to find out the relationships between up-regulated genes an interaction study of all the genes analyzed was carried out. KMEANS clustering algorithm was applied to cluster proteins in the network (*k* = 2). Two main clusters were obtained, a major cluster (green circles) with up-regulated genes and a second minor cluster of down-regulated genes (red circles) (Figures 1, 4). The relationships between the over-expressed genes are shown in Figure 2. The main molecular functions of up-regulated genes (dark green circles) were mainly phosphotransferase activity, insulin-like grown factor receptor and ribonucleoside phosphate binding (Figure 3).

The gene encoding the PRKCB protein is a calcium-activated, phospholipid- and diacylglycerol (DAG)-dependent serine/threonine-protein kinase that is involved in various cellular processes such as B-cell receptor (BCR) signalosome control, oxidative stress-induced apoptosis, androgen-dependent transcription control, insulin signaling, and endothelial cell proliferation.

**Figure 5:**
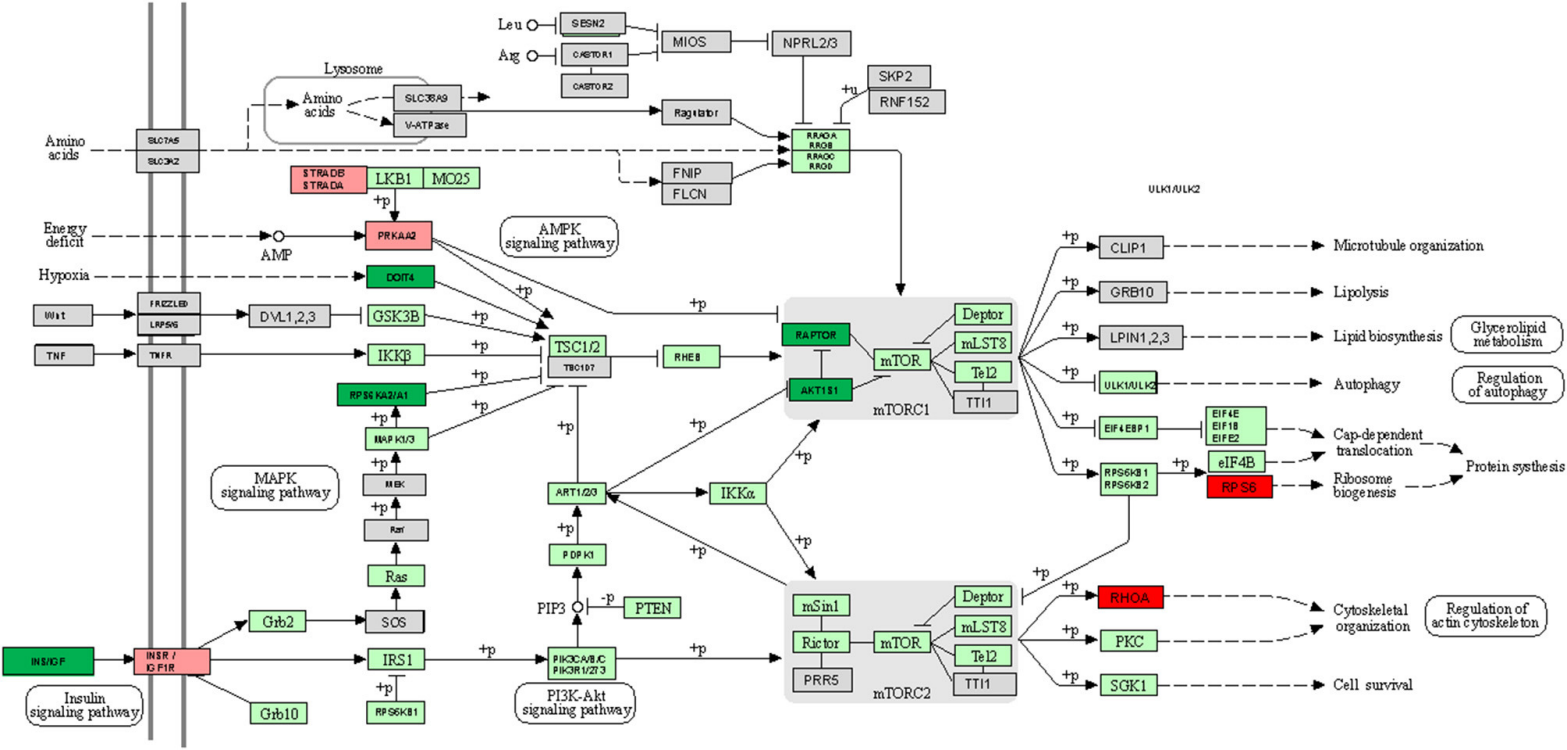
KEGG mTOR signaling pathway map comparing +AMR/+DSA and +DSA vs. –AMR/–DSA KT patients. Green color indicates up-regulated genes. Red color indicates down-regulated genes. The intensity of the color indicates the degree of gene regulation.

The PIK3CG gene encodes aphosphoinositide-3-kinase (PI3K) which finally generates PIP3 which plays a key role activating signaling cascades involved in cell growth, survival, proliferation, motility, and morphology. It links G-protein coupled receptor activation to PIP3 production, involved in immune, inflammatory, and allergic responses. The VEGFC gene encodes a vascular endothelial growth factor C involved in angiogenesis, and endothelial cell growth, stimulating cells proliferation, and migration and also has effects on the permeability of blood vessels. It participates in the maintenance of the differentiated lymphatic endothelial in adults and binds to and activates the KDR/VEGFR2 and FLT4/VEGFR3 receptors. Finally, INS gene encodes a protein that reduces blood glucose concentration. It enhances the permeability of cells toward monosaccharides, amino acids and fatty acids. It accelerates glycolysis, the process of pentose phosphates and the synthesis of glycogen in the kidney. Up-regulated mTOR pathway genes are showed in Figure 5.

### Down-Regulated mTOR Genes in Rejection Kidney Biopsies

The following mTOR pathway genes were down-regulated (≥5-fold) in +DSA/+AMR relative to kidney transplants without rejection (Table 3, Figures 1, 2, 3B). As shown in Figure 2, a total of 9 genes were under-expressed; AKT2, EIF4EBP2, INSR, PRKAA2, RHOA, RPS6, STRADB, VEGFA, and YWHAQ. Of all these genes, the VEGFA was most down-regulated one (>15-fold) and the RPS6 (>10-fold) and RHOA (≥6.5-fold).

With respect to the statistical significance compared to controls after correction, the EIF4EBP2; *P* < 0.005, VEGFA; *P* = 0.002, PRKAA2, *P* = 0.017, RPS6; *P* = 0.012 were significantly down regulated in +AMR biopsies compared with control group. This fact could indicate that the major event is the combination of DSA presence and AMR development. DSA that did not produce AMR development could not influence mTOR gene expression. Down-regulated mTOR genes pathway are shown in Figure 4.

### Interaction Pathway Down-Regulated mTOR Genes in Rejection Kidney Biopsies

In the red cluster, a total of nine down-regulated genes were found. The relationships between the down-regulated genes are shown in Figure 3B. The biological activities that mainly decrease are those related to the proteins encoded by the VEGFA and RPS6 genes (dark red circles). The molecular function of down-regulated gene products (≥5-fold) was also analyzed, and they were mainly protein kinases and carbohydrate derivative binding proteins (Figure 4). The gene that encodes the VEGFA protein is a growth factor active in angiogenesis, vasculogenesis and endothelial cell growth. It induces endothelial cell proliferation, promotes cell migration, inhibits apoptosis, and induces permeabilization of blood vessels. The RPS6 encodes a serine/threonine-protein kinase that is necessary for the mitogenic or stress-induced phosphorylation of the transcription factors CREB1 and ATF1 and for the regulation of the RELA, STAT3, and ETV1/ER81 transcription factors, and that contributes to the activation of the gene by histone phosphorylation and to the regulation of inflammatory genes. A classification of mTOR complex as a function of differential expression between patients with rejection (+DSA/+AMR) and without rejection (+DSA/–AMR and –DSA/–AMR, controls) is shown in Table 4. Finally, cellular processes regulated by mTOR signaling and differential expression genes between patients with rejection (+DSA/+AMR) and without rejection (+ DSA/–AMR and –DSA, controls) are shown in Table 5.

**Table 4:** Classification of mTOR complex as a function of differential expression between patients with rejection (+DSA/+AMR) and without rejection (+DSA/–AMR and –DSA/–AMR).

**mTOR complexes**	**Up-regulated**	**Moderately up-regulated**	**Down-regulated**	**Moderately down-regulated**
mTORC1 Complex	RPTOR	MLST8, MTOR		
mTORC2 Complex		MAPKAP1, MLST8, MTOR, RICTOR		
**mTOR upstream regulators**				
mTORC1 Positive Regulation	IGF1,INS, RPS6KA2	AKT1, AKT2, AKT3, HRAS, IKBKB, IRS1, MAPK1, MAPK3, PDPK1, PIK3C3, PIK3CA, PIK3CB, PIK3CD, PIK3CG, PLD1, PLD2, RHEB, RPS6KA1, RPS6KA5, RRAGA, RRAGB, RRAGC, RRAG, TELO2		INSR
mTORC2 Positive Regulation	RPS6KA2	AKT1, AKT2, AKT3, MAPK1, MAPK, PDPK1, PIK3C3, PIK3CA, PIK3CB, PIK3CD, PIK3CG, RHEB, RPS6KA1, RPS6KA5.		
mTORC1 Negative Regulation	AKT1S1, DDIT4, IGFBP3, PRKAG3,	CAB39, CAB39L, DDIT4L, DEPTOR, FKBP1A, FKBP, PRKAA1, PRKAB1, PRKAB2, PRKAG1, PRKAG2, PTEN, STK11, TP53 (p53), TSC1, TSC2		PRKAA2 STRADB YWHAQ
mTORC2 Positive Regulation:	DDIT4, PRKAG3	CAB39, CAB39L, DDIT4L, DEPTOR, PRKAA1, PRKAB1, PRKAB2, PRKAG1, PRKAG2, STK11, TSC1, TSC2.		PRKAA2 STRADB
**mTOR downstream effectors**				
mTORC1 Positive Regulation	EIF4E, VEGFC.	CHUK, EIF4B, HIF1A, IKBKB, RPS6KB1, RPS6KB2, TP53 (p53), VEGFB	RPS6, VEGFA	
mTORC2 Positive Regulation	PRKCB, PRKCG	AKT1, CDC42, GSK3B, HSPA4, ILK, MYO1C, PRKCA, PRKCE, RPS6KB1, SGK1	RHOA	
mTORC1 Negative Regulation	PPP2CA, ULK1	EIF4EBP1, EIF4EBP2, PPP2R2B, PTPA, TP53 (p53), ULK2.		

**Table 5:** Cellular processes regulated by mTOR signaling and differential expression genes between patients with rejection (+DSA/+AMR) and without rejection (+DSA/–AMR and –DSA/–AMR).

**Cellular processes regulated by mTOR signaling**	**Up-regulated**	**Moderately up-regulated**	**Down-regulated**	**Moderately down-regulated**
Amino acid response		PIK3C3, RRAGA, RRAGB, RRAGC, RRAGD		
Angiogenesis	DDIT4, VEGFC	CHUK, DDIT4L, HIF1A, IKBKB, VEGFB	VEGFA	
Autophagy	ULK1	PIK3C3, ULK2		
Cytoskeletal Organization	PRKCB	CDC42, PRKCA, PRKCE, PRKCG	RHOA	
Growth factor response	HRAS, IGF1, IGFBP3, RPS6KA2	MAPK1, MAPK3, RPS6KA1, RPS6KA5		
Energy stress	PRKAG3	PRKAA1, PRKAB1, PRKAB2, PRKAG1, PRKAG2		PRKAA2
Insulin signaling	INS	AKT1, AKT3, IRS1, PDPK1, PIK3CA, PIK3CB, PIK3CD, PIK3CG, TP53		AKT2, INSR
Lipid metabolism		PLD1, PLD2		
Translation	EIF4E, PPP2CA, RPS6KA2	EIF4B, EIF4EBP1, EIF4EBP2, PPP2R2B, PTPA, RPS6KA1, RPS6KA5, RPS6KB1, RPS6KB2.	RPS6	

## Discussion

In this study, we have analyzed the relationship between mTOR pathway gene expression and histological and immunological changes in humoral rejection biopsies in a large cohort of kidney recipients undergoing transplantation in order to determine if there are differences in the gene expression of the mTOR pathways between +DSA/+AMR and control recipients (–DSA/–AMR, +DSA/–AMR) and its possible influence on transplant outcome.

Genomic quantification of the overall inflammatory burden in the kidney graft seems to be important to determine the suitability of an invasive biopsy. Our results show that several particular gene expressions were increased in biopsies of +DSA/+AMR. Particularly, a higher number of over-expressed genes with mTORC1+ regulation and a number of under-expressed genes with mTORC2+ regulation in biopsies from +DSA/+AMR patients were found.

In this regard, EIF4EBP1 could have an influence on the regulation of protein translation by growth factors and other stimuli that signal through the MAP kinase and mTORC1 pathways. VEGF-A, which has been described to stimulate endothelial cell mitogenesis and cell migration, is also vasodilator and increases microvascular permeability promoting angiogenesis. PRKAA2 is activated in response to cellular metabolic stresses and RPS6 is implicated in cell size regulation and cell proliferation, and similar characteristics occurs in a situation of allograft rejection. The imbalance of gene expression in very important proteins of the mTOR pathway, showed in this study in biopsies, could inhibit mTORC2 activation promoting mTORC1, leading to fibrosis, proliferation and rejection in +DSA/+AMR patients. Thus, a higher number of over-expressed genes with mTORC1+ regulation and a number of under-expressed genes with mTORC2+ regulation in biopsies from +DSA/+AMR patients were found.

Rapamycin's mammalian target (mTOR) is a highly conserved serine/threonine protein kinase and there is much evidence that the mTOR signaling pathway plays a significant role in disease pathogenesis (20, 22, 58). However, changes in RNA levels of the PI3K-AKT-mTOR pathway have not been reported in pathology literature to date. Nevertheless, understanding the burden of inflammation, rejection or accommodation in a graft is critical for the optimization of therapy, following response to chosen interventions and as a means to predict risk stratification for progressive chronic injury and graft loss

Our tested array includes members of the mTORC1 and mTORC2 complexes as well as upstream regulators of many mTOR responses, and downstream genes from the many cellular processes regulated by mTOR complex activation. As a high number of over-expressed genes with mTORC1+ regulation and a number of under-expressed genes with mTORC2+ regulation in biopsies from +DSA/+AMR patients were found, pointing to an important role of particular gene expression of mTOR pathways in kidney rejection. The differential expression of multiple genes in the mTOR signaling pathway in AMR may be an important molecular mechanism that may lead to histological changes suffered by the recipient (59).

However, due to the various components involved in the PI3K-signaling pathway, we are currently unable to study all the components involved in the signal transduction pathway at protein level simultaneously. In this sense, it should be taken into account that when a protein is discovered to be abnormally active it could be due to co-relationships between components involved in the signal transduction pathway. Nevertheless, the PI3K-AKT Signaling PCR Array detects expression of 84 genes in the signaling pathway to obtain a detailed understanding of the molecular profile. The mTOR signaling pathway plays a significant role in immune and inflammatory reactions according to experimental findings (21), It indicates that the increased expression of inflammatory cytokines may be the result of abnormal mTOR activation. In this sense, our group has published the important role of inflammatory cytokines in kidney and liver transplant outcome (4, 48, 60, 61). Logically, a cytokine analysis and real-time correlation analysis would actually prove the association of up-regulated expression of some of mTOR pathway genes. This would pave the way to better management of the rejection episodic kidneys and these analyses will be performed in our following study.

The first generation of mTOR inhibitors do not seem to repress a negative feedback loop that results in the phosphorylation and activation of AKT, whose expression is altered in our study and only inhibits the mTORC1 pathway. These inhibitors may be important in the interpretation of the results of our study, although they are not widely used today. On the other hand, the second generation of mTOR inhibitors are known as ATP-competitive mTOR kinase inhibitors, they inhibit the kinase-dependent functions of mTORC1 and mTORC2 and thus block the feedback activation of PI3K/signaling. AKT, unlike the previous ones that only target mTORC1. Thus, the most important advantage of these second-generation inhibitors is the decrease in phosphorylation of AKT on the blockade of mTORC2 and, also, a better inhibition on mTORC1. Our data does not seem to be affected by this situation due to the same reason mentioned above. Several so-called dual mTOR/PI3K inhibitors (TPdI) have also been developed and are in early-stage preclinical trials and show promising results. Its development has benefited from previous studies with selective PI3K inhibitors. The activity of these small molecules from rapalog activity differs in the way that it blocks both the mTORC1-dependent phosphorylation of S6K1 and the mTORC2-dependent phosphorylation of the AKT Ser473 residue. These inhibitors target PI3K isoforms (p110α, β, and γ) together with the ATP binding sites of mTORC1 and mTORC2 by blocking PI3K/AKT signaling, which may be interesting to study and compare in our future studies more diversified.

Moreover, as there are no important differences in mTOR pathway gene expression in kidney recipients without DSA and with DSA, independently of AMR development, this could indicate that the major event is the particular combination of the DSA presence and AMR development and that the DSAs not producing AMR development could not influence mTOR gene expression. We must study in the future several aspects of the DSA determination as IgG subclasses, complement fixation as C1q or C3d, between others. On the other hand, mTOR pathway expressed genes extracted from peripheral blood rather than from the biopsies would be more appropriate and easier to obtain, unfortunately, we could not determine them in peripheral blood (data not shown).

One of the limitations of our study was the number of patients with kidney rejection in a total of 269 adult sequential kidney transplant (KT) patients recruited during 10 years and analyzed retrospectively. In spite of this, the expression of 84 mTOR pathway genes determined by human mTOR-PCR array technology allowed to analyzed and discriminated effectively the cases of rejection.

Other subject to discuss is whether the mTOR activation was different in various renal diseases and whether mTOR activation could predict relapse with rejection in the baseline renal biopsies of donors in the non-rejection group. This point has been revised in our present study and no special confounding factors have been found (data not shown).

Finally, these findings show the important role of mTOR pathways genes in biopsies with AMR but it is also necessary to quantify inflammation from biopsy tissue, thus providing an important tool for clinical correlation and outcome analysis of kidney transplants. Further research is needed to determine if particular gene expression profiles can prevent graft failure, which highlights the need to develop a more complete understanding of the mechanisms of allograft protection or injury.

## Data Availability Statement

The original contributions presented in the study are publicly available. This data can be found here: 10.6084/m9.figshare.14020901, 10.6084/m9.figshare.14020898.

## Ethics Statement

The studies involving human participants were reviewed and approved by Institutional Review Board and Ethics Committee of the University Clinical Hospital Virgen Arrixaca-Biomedical Research Institute of Murcia (IMIB), Murcia, Spain and conducted in accordance with the Declaration of Helsinki. The patients/participants provided their written informed consent to participate in this study.

## Author Contributions

MM participated in designing the assays, supervising the data generation, analyzing the data, and writing the manuscript. IL, MB, and AP participated in data analysis for gene expression assays and contributed in writing the manuscript. HM-B and RA participated in tissue processing contributed in manuscript writing. VJ-C, AMr, and AMi participated in tissue processing. MM-Q and CB contributed in gene expression data generation, organization, and manuscript writing. SL and JP-M provided the study samples, participated in discussions in data analysis strategies, and manuscript writing. CB and JG were part of the study design and contributed in tissue processing and supervision HLA antibodies optimization, data analysis, and manuscript writing. All authors contributed to the article and approved the submitted version.

## Conflict of Interest

The authors declare that the research was conducted in the absence of any commercial or financial relationships that could be construed as a potential conflict of interest.
